# Recovery of Corneal Innervation after Treatment in Dry Eye Disease: A Confocal Microscopy Study

**DOI:** 10.3390/jcm12051841

**Published:** 2023-02-25

**Authors:** Alberto Barros, Javier Lozano-Sanroma, Juan Queiruga-Piñeiro, Luis Fernández-Vega Cueto, Eduardo Anitua, Ignacio Alcalde, Jesús Merayo-Lloves

**Affiliations:** 1 Instituto Universitario Fernández-Vega, Fundación de Investigación Oftalmológica, Universidad de Oviedo, 33012 Oviedo, Spain; 2 Instituto de Investigación Sanitaria del Principado de Asturias (ISPA), 33011 Oviedo, Spain; 3 Department of Surgery and Medical-Surgical Specialities, Universidad de Oviedo, 33006 Oviedo, Spain; 4 Biotechnology Institute (BTI), 01007 Vitoria, Spain

**Keywords:** corneal nerves, corneal confocal microscopy, dry eye disease

## Abstract

Purpose: To analyze the changes in corneal innervation by means of in vivo corneal confocal microscopy (IVCM) in patients diagnosed with Evaporative (EDE) and Aqueous Deficient Dry Eye (ADDE) and treated with a standard treatment for Dry Eye Disease (DED) in combination with Plasma Rich in Growth Factors (PRGF). Methods: Eighty-three patients diagnosed with DED were enrolled in this study and included in the EDE or ADDE subtype. The primary variables analyzed were the length, density and number of nerve branches, and the secondary variables were those related to the quantity and stability of the tear film and the subjective response of the patients measured with psychometric questionnaires. Results: The combined treatment therapy with PRGF outperforms the standard treatment therapy in terms of subbasal nerve plexus regeneration, significantly increasing length, number of branches and nerve density, as well as significantly improving the stability of the tear film (*p* < 0.05 for all of them), and the most significant changes were located in the ADDE subtype. Conclusions: the corneal reinnervation process responds in a different way depending on the treatment prescribed and the subtype of dry eye disease. In vivo confocal microscopy is presented as a powerful technique in the diagnosis and management of neurosensory abnormalities in DED.

## 1. Introduction

The cornea is one of the most densely innervated tissues in the human body, mainly by sensory and autonomic nerve fibers. In addition to the importance of its sensory functions, corneal nerves help maintain the functional integrity of the ocular surface by releasing trophic substances that promote epithelial homeostasis and by activating circuits in the brainstem that stimulate tear production and blinking [1]. Damage to these nerve endings, whether mechanical in the case of eye surgery, or caused by ocular and systemic diseases, can lead to long-term damage to the integrity of the ocular surface [1,2].

Dry Eye Disease (DED) is one of the most common diseases in ocular surface consultationss worldwide [3], and it is accompanied by discomfort or pain sensations [4]. The Tear Film and Ocular Surface Society (TFOS) Dry Eye Workshop (DEWS) II [4] defined DED as a multifactorial disease of the ocular surface characterized by a loss of homeostasis of the tear film, and accompanied by ocular symptoms, in which tear film instability and hyperosmolarity, ocular surface inflammation and damage, and neurosensory abnormalities play etiological roles [4]. Two main subtypes of dry eye have been described [5]. In evaporative dry eye (EDE), tear hyperosmolarity is the result of excessive tear film evaporation in the presence of normal tear function, while in aqueous deficient dry eye (ADDE) hyperosmolarity is due to reduced tear secretion in the presence of a normal evaporation rate [6].

There is a growing interest in the study of neurosensory alterations of corneal innervation related with DED [6]. In vivo corneal confocal microscopy (IVCM) is a safe and noninvasive technique for the study and analysis of corneal innervation. Recently, several studies have used IVCM to analyze changes in the subbasal nerve plexus in DED [7,8,9], and also to evaluate the differences in this innervation between different types of dry eye [10,11]. This technique has been previously used to analyze corneal innervation in different ocular conditions, such as corneal dystrophies [12], trauma [13], infections [14], or in systemic diseases such as diabetes [15,16]. More recently, IVCM has been used to evaluate changes in the subbasal nerve plexus of patients affected by neurodegenerative diseases, such as Fibromyalgia [17,18], Parkinson’s disease [19,20], Multiple sclerosis [21,22,23] and even to evaluate small fiber neuropathy after viral infection with Sars-CoV-2 [24].

DEWS II established treatment strategies for DED to address tear film insufficiency (artificial tears), alterations of the palpebral margin (lid hygiene), inflammation (corticosteroids [25] immunosuppressants and immunomodulators such as Cyclosporine A, Tacrolimus and Lifitegrast [26]). Surgical approaches and nutritional supplements (diets, vitamin supplements) are also used to treat DED [27].

Artificial tears composed of blood derivatives have been gaining prominence in the treatment of DED, most notably Autologous Serum (AS), which was the first derivative of a patient’s own blood to be used for the treatment of DED [28,29]. In recent years, a type of blood-based eye drops has been described, known as PRGF–Endoret^®^ (BTI, Vitoria, Spain), which is a plasma rich in growth factors, including epidermal growth factor (EGF), transforming growth factor–β1(TGF-β1), platelet-derived growth factor (PDGF), insulin-like growth factor I (IGF-I), vascular endothelial growth factor (VEGF), nerve growth factor (NGF), and fibronectin among others. In contrast to AS, PRGF–Endoret^®^ is formulated without inflammatory cells such as leukocytes [30,31]. PRGF treatments have shown regenerative effects on the ocular surface epithelium in neurotrophic keratitis [32], persistent epithelial defects [33], and postoperative processes [34,35]. In addition, the safety and efficacy of PRGF for the treatment of DED have been previously demonstrated [36,37] and a clinical trial using PRGF–Endoret^®^ compared with AS in the treatment of moderate and severe dry eye is currently under way, with results expected in 2023 [38].

However, little is known about the effect of PRGF treatment on the recovery of corneal innervation.

The purpose of this study was to analyze the changes in corneal innervation in patients diagnosed with evaporative and aqueous deficient dry eye and treated with a standard treatment for dry eye disease in combination with plasma rich in growth factors.

## 2. Methods

This observational retrospective, longitudinal study was conducted in accordance with the Declaration of Helsinki and approved by the Committee on Ethics in Medical Research of the Principality of Asturias with the code number 2022.167 of 11 May 2022. It follows the Strengthening the Reporting of Observational Studies in Epidemiology (STROBE). Prior to data collection, patients were informed about the purpose of the study and the procedures were read and signed by them.

Eighty-three patients with a diagnosis of DED were recruited in this study. Inclusion criteria were Ocular Surface Disease Index (OSDI) score over 13, indicating moderate grade or higher of the disease, and tear break up time less than 10 s.

In addition, subjects who had a Schirmer’s test of less than 5.5 mm were included in the subgroup of predominantly aqueous deficient dry eye (ADDE), while those above 5.5 mm were included in the predominantly—evaporative dry eye group (EDE) [6,39].

Other eye diseases that could indirectly affect corneal integrity were exclusion criteria (glaucoma, macular degeneration, previous ocular surgery procedures or corneal infections such as herpes virus, bacterial or fungal keratitis, adenovirus, and Acanthamoeba). Patients presenting systemic diseases that could cause alterations in corneal innervation such as Diabetes, Fibromyalgia, Parkinson, dysautonomia, etc. were also excluded.

### 2.1. In Vivo Confocal Microscopy (IVCM)

Patients were examined using a Heidelberg^®^ Retina Tomograph III confocal microscope equipped with the Rostock Cornea Module (Heidelberg Engineering. Heidelberg, Germany) with a 670 nm wavelength Helium-Neon diode laser, 63x objective and numeric aperture of 0.9.

Before the beginning of the examination with the IVCM, topical anesthetic (Tetracaine 0.1% + Oxibuprocaíne; Alcon Cusí) was applied to the eye and a sterile cap (TomoCap^©^, Heidelberg Engineering) was attached to the lens of the microscope and a high viscosity gel (Recugel^®^, Baush & Lomb^®^, Vaughan, ON, Canada) was used as a bonding agent between the cap and the lens.

One eye of each patient was randomly selected (randomizer.org, accessed on 6 September 2022) and images of the corneal nerves of the selected eye were taken using the section mode in the central and paracentral cornea.

A total of 15 to 25 images of five non overlapping areas of each eye were selected.

The examination addressed the analysis of the entire corneal thickness from the epithelium to the anterior stroma, in order to make sure that the level of the sub basal nerve plexus was swept. The size of the images obtained was 384 × 384 pixel, which corresponds to an area of 400 × 400 µm.

The images were analyzed and quantified automatically with ACCMetrics^©^ software (MA Dabbah, Imaging science and Biomedical engineering, Manchester, UK) [40,41].

**Figure 1:**
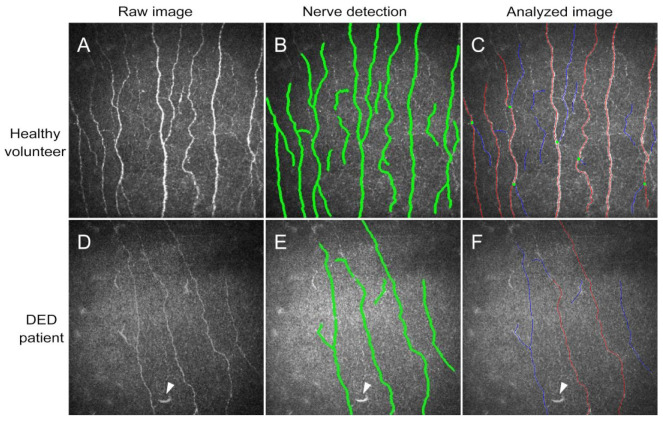
Examples IVCM image analysis using ACCMetrics^©^ software. (**A**–**C**) show an image from a healthy cornea. (**D**–**F**) show the analysis process in the cornea of a DED patient. Note the differences in nerve fiber number and thickness between healthy (**A**) and DED (**D**) raw images (as obtained directly from the microscope). ACCMetrics^©^ software detects nerve fibers (traces marked in green in (**B**,**E**) automatically and segregates traces in primary axons (red), first branches (blue) and the points of branching (green dots) in the analyzed images for quantification((**C**,**F**). Arrowheads in (**D**–**F**) show one dendritic cell in DED patient images.

This software reported the measurements of the following seven parameters: Corneal Nerve Fiber Density (CNFD), which shows the total number of nerves per mm^2^; Corneal Nerve Branch Density (CNBD), the number of first branches originating from primary axons per mm^2^; Corneal Nerve Fiber Length (CNFL), which measures the total length of all nerve fibers and branches in mm/mm^2^; Corneal Total Branch Density (CTBD), the total number of branches/mm^2^; Corneal Nerve Fiber Area (CNFA) in mm^2^/mm^2^; Corneal Nerve Fiber Width (CNFW), which shows the average nerve fiber width of the sub basal plexus in mm/mm^2^, and Corneal Nerve Fractal Dimension (CNFrD), which is an indicator of the structural complexity of the corneal nerve image (Figure 1).

The same images were also analyzed using the cell counter plugin of FIJI^©^ image analysis software (ImageJ 1.53c: NIH, Bethesda, MD, USA) with the objective of quantify the incidence of neuromas (total number of neuromas per frame), beaded axons (count of the number of nerves which presented beaded axons per frame), and number of dendritic cells per frame of the central cornea. This part of the measurements was carried out in a semi-automatic way, in which the operator marked the presence of each of the disturbances in each image, and the Cell Counter plugin automatically calculated the total numbers [42].

The images were analyzed by a single experienced researcher. The final value used for each parameter was the average of the measure of all selected images of each patient.

To avoid any mistake in the classification of selected IVCM morphological alterations, once a pathological sign of the three items mentioned previously was located, the corneal structure was examined in detail with the aim of differentiating them from similar anatomical structures [43,44].

Both types of analysis with ACCMetrics^®^ and ImageJ^®^ software were applied to each of the images from all patients. The average of the values obtained for each parameter were used for statistical analysis.

### 2.2. Tear Film Break Up Time

To assess tear film stability, a drop of preservative-free 2% sodium fluorescein was instilled into the lower fornix of the patient’s eye. The eye was then observed at the slit lamp at low magnification and the patient was urged to blink several times and keep the eyelids open until dark areas were observed within the green staining provided by the fluorescein. The time between the last blink and the appearance of the dark areas was recorded.

### 2.3. Schirmer Test

To quantify tear production, the Schirmer test (Katena^®^, Denville, NJ, USA) was performed on all patients by placing the paper strips on the temporal part of the inner edge of the lower eyelid for 5 min after instillation of a drop of topical anesthetic to minimize reflex tearing [45,46]. After 5 min, the millimeters of impregnated strip were measured.

### 2.4. Diagnostic Questionnaires

The presence of symptoms of ocular surface disease as well as their perceived severity, were assessed with the Ocular Surface Disease Index (OSDI) and the severity and intensity scales of the Symptoms Analysis in Dry Eye (SANDE).

### 2.5. Treatment

The patients were divided into two groups according to the treatment therapy prescribed by the ocular surface unit of Fernández-Vega Ophthalmological Institute.

Thirty-two patients were treated with a DED treatment therapy consisting of a corticosteroid regimen with Fluorometholone 0.1% (FML^®^, Allergan©) in a descending pattern of 4 times a day for one week, three times a day for one week, twice a day for one week and once a day for one week, combined with ocular surface hydration with Trehalose 3% and sodium hyaluronate 0.15% (Thealoz Duo^®^, Thea, Milan, Italy) 4 times a week and eyelid hygiene once a day, both until the follow-up visit. This group was named as the standard treatment group.

Fifty-one patients were treated with the same treatment therapy as the previous group, plus an ocular surface regeneration treatment consisting of Plasma Rich in Growth Factors 4 times a day until 3 months.

According to manufacturer’s instructions, blood from this treatment group was collected into 9-mL tubes with 3.8% (*wt*/*v*) sodium citrate or in serum collection tubes (Z Serum Clot activator, Vacuette, GmbH, Kremsmünster, Austria). Blood samples were centrifuged at 580× *g* for 8 min at room temperature in an Endoret System centrifuge (BTI Biotechnology Institute, S.L., Miñano, Alava, Spain); the whole plasma column over the buffy coat was collected using Endoret ophthalmology kit (BTI Biotechnology Institute, S.L., Miñano, Alava, Spain) avoiding the layer containing leukocytes. Platelets and leukocytes counts were performed with a hematology analyzer (Micros 60, Horiba ABX, Montpellier, France). Plasma preparations were incubated with Endoret activator (BTI Biotechnology Institute^©^, S.L., Miñano, Alava, Spain) at 37 °C for 1 h and PRGF supernatants were filtered, aliquoted and stored at 80 °C until use. All procedures were performed under sterile conditions inside a laminar flow hold. The patients were instructed to keep the PRGF eye drops dispensers at −20 °C for a maximum of 3 months [47,48,49]. This group was named PRGF treatment group.

All patients repeated the same tests at the follow-up visit.

### 2.6. Statistical Analysis

The SPSS statistical software v. 22 for Windows (SPSS Inc., Chicago, IL, USA) was used for the analysis of the data. Values were expressed as mean ± standard error of the mean (SEM). Normality of the sample was checked with the Kolmogórov–Smirnov and Shapiro Wilk tests according to the sample size. The Student’s *t*-test and the Wilcoxon test were used to compare the means of the different study variables of paired samples according to the distribution of the data. In addition, the effect size was calculated with Cohen’s *d* for each of the variables in all study groups.

## 3. Results

This observational, longitudinal, and retrospective study involved 83 patients with diagnosis of Dry Eye Disease who have visited the Fernández-Vega Ophthalmological Institute between January 2020 and April 2022. Table 1 shows the demographic data; no statistically significant differences were found from the inclusion criteria for both groups.

### 3.1. General Results

#### 3.1.1. Corneal Nerve Quantification

As shown in Table 2, the automatic analysis of corneal nerves using ACCMetrics^®^ software showed that the morphology of the subbasal nerve plexus was not significantly altered in the standard group. The data revealed an increase in nerve branching (CNBD and CTBD) and fractal dimension (CNFrD), which was not significant in any of the parameters. The rest of the values extracted from the automatic analysis such as CNFD, CNFL, CNFW, CNFA saw slightly decreased values at follow-up with respect to the baseline visit, without being statistically significant for any of the morphological parameters.

The PRGF treatment group showed an evident increase in CNFD and CNFL (*p* < 0.001), CNBD, CNFA and Fractal Dimension (*p* < 0.005) and CTBD (*p* < 0.05).

Corneal Nerve Fiber Width did not show any differences.

#### 3.1.2. Morphological Alterations and Cell Infiltration

The semi-automatic analysis of IVCM images using FIJI^®^ software showed a significant reduction in the presence of dendritic cells for the standard treatment group (*p* < 0.05), as well as in the count of Axonal Beads for both groups (*p* < 0.005 for standard treatment group and *p* < 0.05 for PRGF treatment group).

Although the presence of neuromas was low in both groups, the changes observed were not significant after treatment.

#### 3.1.3. Tear Film and Ocular Surface Disease Questionnaires

The assessment of tear quantity with the Schirmer test did not show statistically significant differences in either treatment group at the follow-up visit. Tear stability measured with the Fluorescein Break Up Time was significantly increased in the PRGF treatment group (*p* < 0.005) compared to the standard treatment group (*p* = 0.654).

The OSDI score decreased significantly for both the standard treatment group (*p* < 0.005) and the PRGF combination therapy group (*p* = 0.005).

The SANDE frequency and severity questionnaire score were also significantly decreased for both treatment groups (*p* < 0.001).

### 3.2. Subtypes of Dry Eye Disease

To study the response of corneal innervation according to the type of dry eye, the sample was divided into EDE and ADDE according to the measurement of tear quantity.

#### 3.2.1. Difference between Subtypes of Dry Eye Disease

Baseline data were compared to identify differences between the two subtypes of dry eye disease studied. The CNFD values for the ADDE subtype was 15.007 ± 8.122 fibers per mm, while for the EDE group it was 18.218 ± 5.99 (*p* = 0.066). CNBD was 18.766 ± 16.323 branches per mm in the ADDE and 21.129 + 13.541 (*p* = 0.339) for the evaporative subgroup. For CNFL the values for the ADDE were 10.784 ± 4.228 mm per mm, and 12.185 ± 3.130 (*p* = 0.147) for the EDE. Differences were also not statistically significant for both groups in neuromas (*p* = 0.195), dendritic cells (*p* = 0.700) and axonal beadings (*p* = 0.861). For the ADDE group, the psychometric questionnaires showed an OSDI of 41.337 ± 23.638, SANDE frequency of 65.943 ± 28.673 and SANDE intensity of 60.188 ± 25.850, while for the EDE group the values were OSDI 38.400 ± 20.648 (*p* = 0.748), SANDE frequency 71.142 ± 26.297 (*p* = 0.326) and SANDE intensity of 65.535 ± 21.745 (*p* = 0.420).

#### 3.2.2. Evaporative Dry Eye Subtype

Fifteen patients of this subtype of Dry Eye were treated with PRGF while fourteen patients were treated with the standard treatment group.

Baseline values were compared according to the assigned treatment, finding no significant differences for any of the treatment groups (*p* = 0.363 for CNFD, *p* = 0.692 for CNBD, *p* = 0.233 for CNFL, *p* = 0.234 for Schirmer, *p* = 0.870 for Fbut, *p* = 0.847 for dendritic cell count, *p* = 0.477 for neuromas count, *p* = 0.533 for Beadings count, *p* = 0.486 for OSDI score, *p*= 477 for neuromas count, *p* = 0.533 for Beadings count, and *p* = 0.062 for SANDE intensity questionnaire) apart from the SANDE frequency questionnaire which showed significant differences in baseline values according to treatment groups (*p* = 0.027).

**Figure 2:**
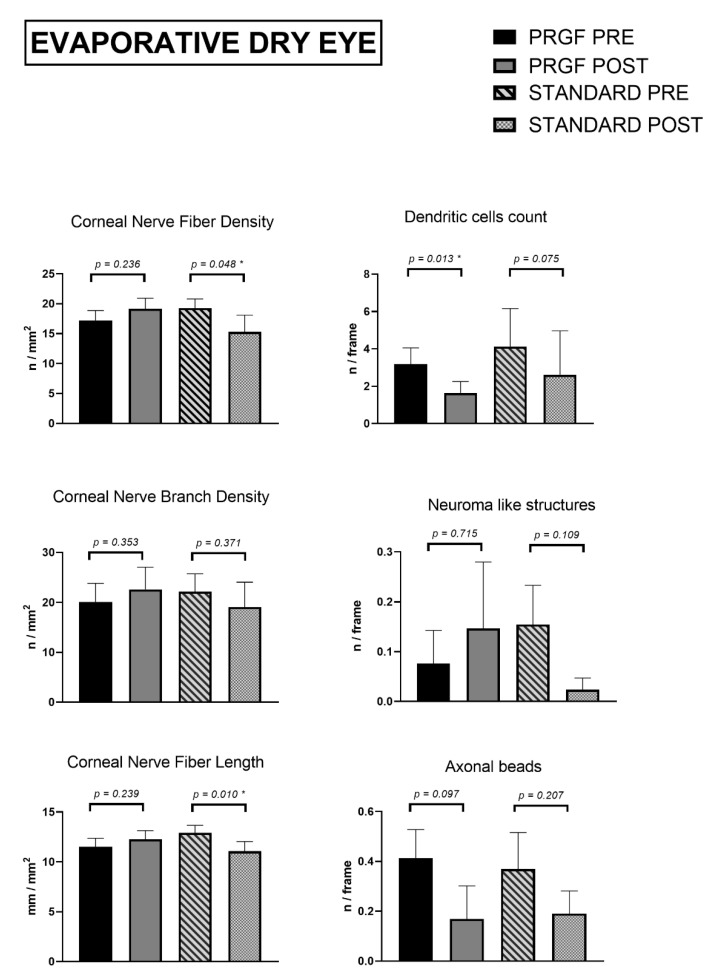
Corneal nerve quantification and morphological alterations Evaporative Dry Eye subtypes according to groups of treatment. Asterisks (*) in the graphs indicate statistical differences between groups (*p* < 0.05).

As shown in Figure 2, nerve parameters such as density, length and number of branches increased slightly in the PRGF-treated group at the follow-up visit but were not statistically significant. The same values decreased for the standard treatment group, being significant for CNFD (*p* < 0.05) and for CNFL (*p* = 0.01).

The morphological alterations of nerves (neuromas and beadings) and cell infiltration (dendritic cells) showed no relevant differences in any of the parameters analyzed, apart from the dendritic cell count in the PRGF treatment group (*p* < 0.05). Although not significant, a certain increase in the presence of neuromas was observed at the follow-up visit for the PRGF treatment group.

**Figure 3:**
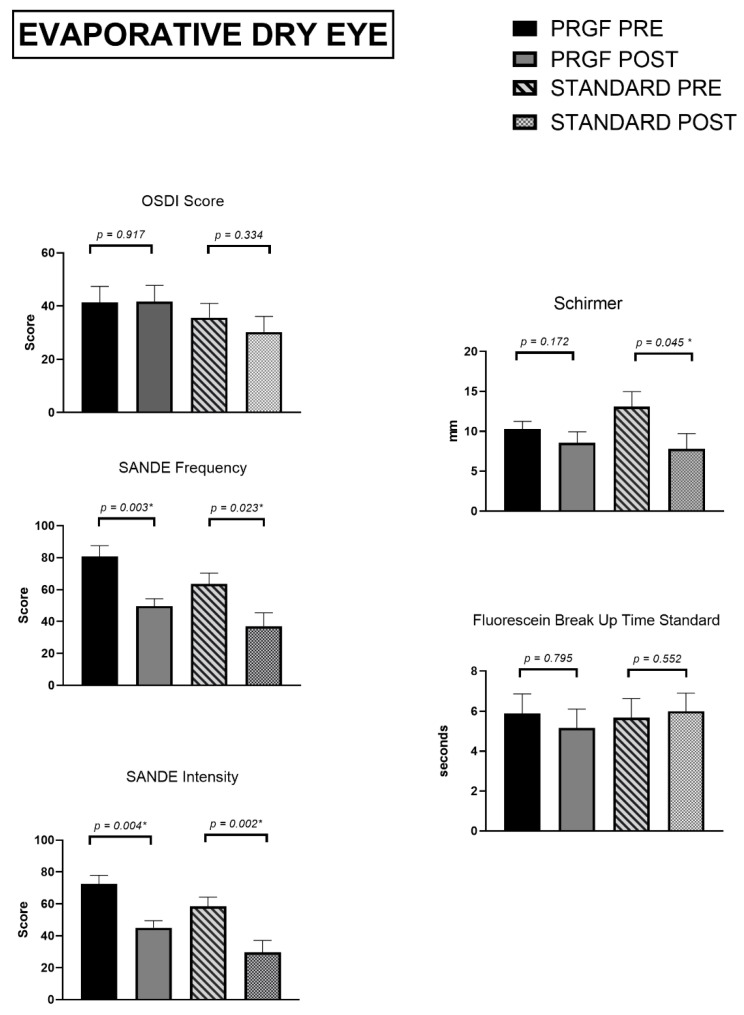
Tear film and psychometric questionnaires for Evaporative Dry Eye Subtype. Asterisks (*) in the graphs indicate statistical differences between groups (*p* < 0.05).

Focusing on the tear film and psychometric questionnaires, results in Figure 3 showed that tear film volume was reduced was reduced for both treatment therapies, with the reduction being statistically significant for the standard treatment group (*p* = 0.045).

Tear break-up time did not change significantly for either treatment group at the follow-up visit.

The OSDI questionnaire remained significantly unchanged at the follow-up visit in both the standard and combined PRGF treatment groups.

For the SANDE frequency and intensity questionnaires, reductions were significant for both groups of treatment at the follow-up visit (*p* < 0.005).

#### 3.2.3. Aqueous Deficient Dry Eye Subtype

Thirty-three patients of this subtype of Dry Eye were treated with PRGF while eighteen patients were treated with the standard treatment group. Data for one patient in the PRGF treatment group was estimated as a missing value because no post-treatment tear volume value was available.

As in the EDE subtype, baseline values were compared according to treatment type and no significant differences were found for CNBD (*p* = 0.148), FBut (*p* = 0.749), Schirmer (*p* = 0.232), dendritic cell count (*p* = 0.916), neuromas (*p* = 0.619), OSDI score (*p* = 0.286), SANDE frequency (*p* = 0.798) and SANDE intensity (*p* = 0.250). However, significant differences in these values were identified for CNFD (*p* = 0.001), CNFL (*p* = 0.003) and axonal beads count (*p* = 0.013).

**Figure 4:**
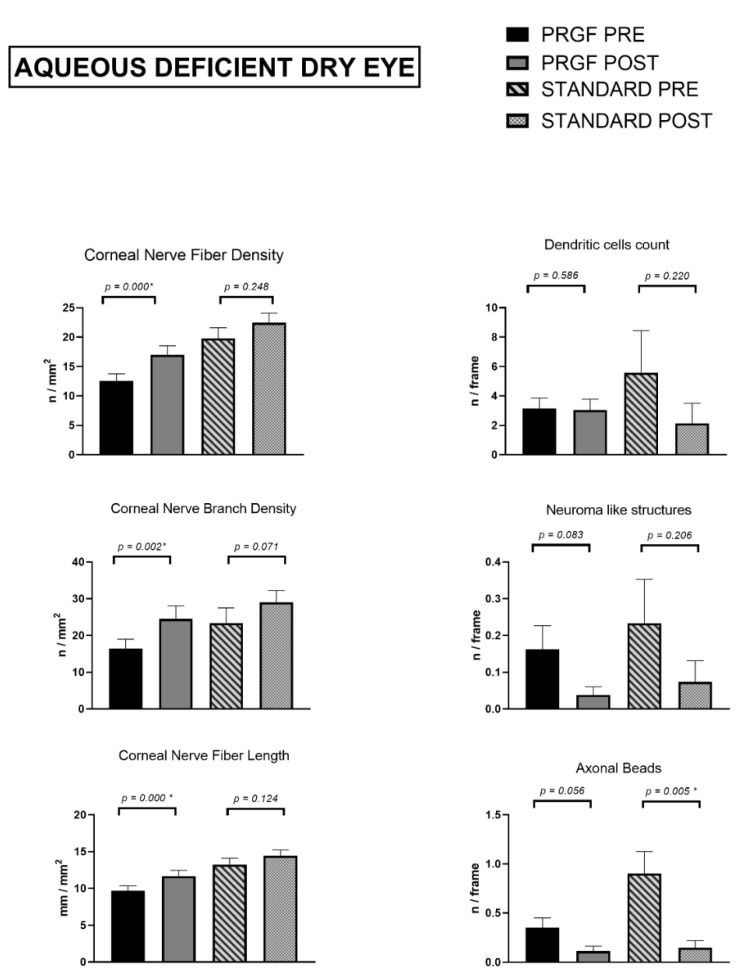
Corneal nerve quantification and morphological alterations ADDE subtype according to groups of treatment. Asterisks (*) in the graphs indicate statistical differences between groups (*p* < 0.05).

In this subtype of DED, Figure 4 shows a statistically significant increase for length, density, and number of nerve branches (*p* < 0.05 for all of them) after combined treatment with PRGF, compared to the standard treatment group, which also showed an increase, without statistical significance.

In the case of morphological alterations and presence of inflammation, axonal beads significantly decreased in the standard treatment group (*p* = 0.05). No other relevant differences were observed in any of the parameters analyzed, although their values decreased at the follow-up visit in both treatment subgroups.

**Figure 5:**
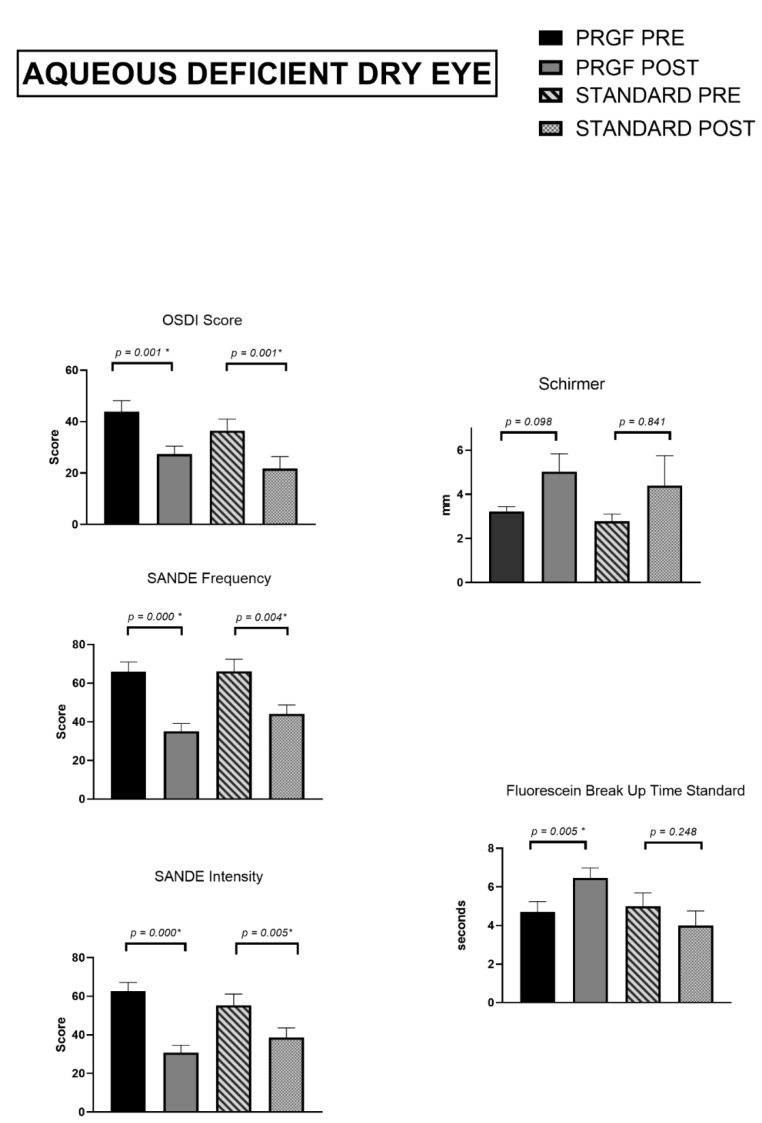
Tear film and psychometric questionnaires for Aqueous Deficient Dry Eye. Asterisks (*) in the graphs indicate statistical differences between groups (*p* < 0.05).

The tear film study showed (Figure 5) an increase for the combined PRGF treatment subgroup and the standard treatment subgroup, but this was not significant for either of them.

An increase in tear break-up time was found in the PRGF subgroup (*p* = 0.005) while the same value decreased slightly for the standard treatment subgroup (*p* = 0.248).

As for the psychometric questionnaires, both OSDI and SANDE frequency and intensity had significantly reduced values at the follow-up visit, with *p* < 0.005 for each of them in both treatment subgroups.

### 3.3. Effect Size

To answer the question of how big the change in the analyzed variables after each of the treatments was, the effect size was calculated for each of them according to the subtype of DED, comparing the values obtained at the baseline visit with those at the follow-up visit. (Table 3).

For the EDE subtype, the standard treatment had a negative effect for CNFD, CNBD and CNFD, and for nerve length was at the edge of the medium size. With PRGF treatment, the effect size, although low, was positive for the same variables.

As for morphological alterations of the subbasal nerve plexus in this type of DED, the effect size was negative for all of them, apart from neuromas in the PRGF treatment group, although with a low effect. The effect size value was at the limit of the medium consideration for dendritic cells in the PRGF treatment group.

The tear film study did not reveal a significant effect size on tear break-up time. However, a negative effect of medium size on tear quantity—quantified by Schirmer’s test—was observed for the standard treatment group.

No significant effect size was observed in both treatment groups for the OSDI score, while for the SANDE questionnaires this value was medium-high for both groups and higher for the PRGF treatment group.

In the aqueous deficient dry eye subtype, the two treatment groups had a positive effect on CNFD, CNBD and CNFL, with Cohen’s d being higher for the PRGF treatment group for these three variables. The effect was negative for all of the three subbasal nerve plexus morphological alterations studied, with a value of 0.756—medium-high effect size—for axonal bead count in the standard treatment group.

Tear analysis for this dry eye subtype showed higher Cohen’s d values in the PRGF treatment group. Note the negative effect for BUT in the standard treatment group.

The effect size was medium for the OSDI score in both treatment groups and for the SANDE questionnaires in the standard treatment group, while in the PRGF treatment group the effect found was high.

## 4. Discussion

We conducted a study including 83 eyes of 83 patients diagnosed with DED to compare the changes in corneal innervation when treated with a standard treatment and the same treatment in combination with plasma rich in growth factors.

The results of the study suggested that the combined treatment therapy with PRGF outperforms the standard treatment therapy in terms of subbasal nerve plexus regeneration, significantly increasing length, number of branches and nerve density, as well as significantly improving the stability of the tear film analyzed with fluorescein BUT.

Our findings were consistent with previous studies analyzing the response of corneal innervation to different topical treatments.

The positive effect of hematic derivatives on the ocular surface has been studied repeatedly. Fox et al. [28] described in 1984 an improvement in the symptomatology and objectivity of fifteen patients treated with artificial tears made from the patient’s serum, which had not improved with conventional artificial tears. These results were also confirmed by other authors, including a randomized clinical trial [50], which is consistent with our results in a significant decrease in OSDI, improvement in BUT and also found no significant changes in the Schirmer’s measurement.

The effect that hematic derivatives induce in corneal innervation has also been the subject of previous studies. In this regard, there has been some discrepancy between publications. Giannaccare et al. [51] found a significant increase in CNFD, CNFL and CNFrD in patients treated with peripheral allogenic blood serum and umbilical cord blood serum in their prospective study, although with a short follow-up period. On the other hand, Mahelkova’s prospective work [52] found no differences in subbasal plexus nerve fibers in patients treated with autologous serum tears. These discrepancies may be explained by the difference in measuring devices, the cause of the DED, the sample size or the type of hematic derivative.

PRGF–Endoret^®^ is an autologous platelet plasma rich in growth factors, standardized to reduce the proinflammatory cytokines in its formulation by removing leukocytes and by a heating treatment [53]. This feature would help to treat DED, as it is associated with a chronic inflammatory process [54].

Due to the high concentration in growth factors, PRGF-based eye drops promote a range of biological events, including cell proliferation, migration, and differentiation, while protecting against microbial contamination on the ocular surface [47,55,56]. A higher concentration of most growth factors was found in PRGF formulations than in AS [57]. Among growth factors, NGF has been shown to stimulate corneal epithelium proliferation and promote subbasal nerve plexus regeneration [58].

Our results showed no statistically significant differences in the subbasal nerve plexus between the two DED subtypes analyzed, with smaller values for ADDE. Other studies found significant reductions in corneal nerves in ADDE versus EDE using a semi-automated quantification method that reports higher values for these parameters than we obtained with the technique used in our study [10]. However, when we grouped our sample into the two dry eye subtypes according to the treatment prescribed, we found significant differences in the baseline values of CNFD and CNFL of the ADDE, with lower values in the subgroup of combined treatment with PRGF. This treatment subgroup also had higher OSDI and SANDE values and previous studies found a negative correlation between OSDI and CNFD [59].

This may be explained by the fact that in professional practice within the ocular surface unit, the ophthalmologist could have prescribed combined treatment with plasma rich in growth factors to patients who showed a higher subjective severity as measured by psychometric questionnaires, based on his previous experience and research in this field [32,35,55,60].

On the other hand, we found no significant changes in corneal innervation at the follow-up visit in the standard treatment group, and even a slight decrease in CNFL and CNFD. There are some discrepancies between previous studies regarding how corticosteroid [61,62] and immunomodulation-based treatments [63,64] affect the subbasal nerve plexus in DED. Reduction in subbasal nerves, as seen in cases of DED treated with cyclosporine A [64], may be explained by reduced NGF production and other cytokines, such as IL-1 and TNF-α. Although it is known that these treatments act by intervening in the inflammatory process associated with the disease, their mechanisms of action on corneal innervation remains unclear.

Consistent with a reduction of inflammation, we observed a significant reduction in the dendritic cell count in the standard treatment group. Villani et al. [62] also found no significant changes in the subbasal nerve plexus in their open-label and masked study, using a semi-automated nerve fiber quantification system. This study and that of Li Bei [65] are also consistent with ours in the reduction of dendritic cells after treatment with topical steroids.

The DEWS established at its first meeting in 2007 that hyperosmolarity and tear film instability are the starting point for the development of dry eye disease [66] The main cause of EDE is known to be tear film disruption accompanied by Meibomian gland dysfunction, and reduced tear secretion secondary to age-related degeneration of the lacrimal gland is the main cause of lacrimal secretion deficit dry eye [6]. It is therefore difficult to draw the line between dry eye due to a lack of secretion and evaporative dry eye, so it would be more accurate to talk about which is the predominant category in each case [5].

Our results suggest that the combined treatment with PRGF in DED contributes to the creation of the ocular surface regeneration scenario, in which corneal regeneration is promoted [3,67]. When this regenerative scenario occurs in ADDE, it induces an improvement in the stability and quantity of the tear film, as well as anti-inflammatory agents and growth factors, which create the perfect context for the corneal reinnervation process, and we observed a significant increase in this process compared to the group treated with standard treatment.

However, when the scenario occurs in EDE, the treatment also contributes to the regeneration of the ocular surface, but without solving one of the main problems which is the alteration of the eyelids and dysfunction of the Meibomian glands, which will continue to generate a situation of evaporative excess and instability of the tear film, which are described as one of the main pathogenic factors of the vicious circle in ocular surface disease [67].

As a limitation of the study, in the analyzed sample, all the patients were prescribed for eyelid hygiene protocol, but the condition of the eyelids was not monitored, so it could not be concluded whether the alterations at this level were resolved at the follow-up visit. This may explain why the most significant changes at the level of the sub basal nerve plexus are in the ADDE and raises the need for future studies quantifying the status of the eyelids and the degree of Meibomian gland dysfunction. Moreover, all the patients included in our study had a BUT below 10 s, so all had an evaporative component, and were subsequently grouped according to the reduction in tear secretion, so our study is limited by not having subjects with BUT greater than 10 s accompanied by low Schirmer values. The sample size, although in line with other published studies, can be considered small and unrepresentative and our study is also limited in this aspect, so prospective studies with larger samples are needed.

## 5. Conclusions

The corneal reinnervation process responds in a different way depending on the treatment prescribed and the subtype of dry eye disease. This process can be monitored and quantified non-invasively and in vivo using confocal microscopy, which is presented as a technique that can be useful in the diagnosis and management of one of the five main pathogenic factors of ocular surface diseases, of which DED is one of the most important; this can contribute to personalized treatment therapies for the disease.

## Figures and Tables

**Table 1:** Demographics and inclusion criteria.

	Standard Treatment		PRGF Treatment		
	n/Mean	±SEM	n/Mean	±SEM	*p* Value
Number of subjects	32	-	51	-	-
Age	55.13	2.08	56.08	2.29	0.766
Sex distribution (female/male)	23/9	-	39/12	-	0.639
Previous OSDI Score	36.12	3.38	43.17	3.52	0.219
Previous Break Up Time	5.27	0.58	4.93	0.47	0.581
Time of treatment (months)	4.75	0.62	4.80	0.55	0.621

**Table 2:** General results.

	Standard Treatment				PRGF Treatment			
	Baseline	Follow-Up			Baseline	Follow-Up		
	Mean (±SEM)	Mean (±SEM)	*p* Value	Effect Size	Mean (±SEM)	Mean (±SEM)	*p* Value	Effect Size
OSDI SCORE	36.119 ± 3.386	25.456 ± 3.680	0.002*	0.412	43.169 ± 3.520	31.553 ± 2.926	0.005 *	0.366
SCHIRMER (mm)	7.193 ± 1.277	5.875 ± 1.159	0.194	0.136	5.340 ± 0.561	6.125 ± 0.738	0.685	0.121
FBut (sec)	5.269 ± 0.588	4.875 ± 0.595	0.654	0.087	4.935 ± 0.468	6.106 ± 0.463	0.003 *	0.262
SANDE Frequency	65.000 ± 4.512	41.093 ± 4.455	0.000*	0.666	70.102 ± 4.196	39.591 ± 3.263	0.000 *	0.820
SANDE Severity	56.719 ± 4.113	34.687 ± 4.312	0.000*	0.654	65.510 ± 3.557	35.102 ± 3.142	0.000 *	0.915
CNFD (n/mm^2^)	19.572 ± 1.188	19.340 ± 1.615	0.859	0.020	13.827 ± 1.019	17.556 ± 1.162	0.000 *	0.338
CNBD (n/mm^2^)	22.874 ± 2.728	24.672 ± 2.925	0.432	0.079	17.440 ± 2.092	23.796 ± 2.762	0.002 *	0.257
CNFL (mm/mm^2^)	13.087 ± 0.590	12.965 ± 0.677	0.832	0.024	10.177 ± 0.537	11.863 ± 0.603	0.000 *	0.293
CTBD (n/mm^2^)	38.181 ± 3.751	41.022 ± 4.118	0.466	0.190	32.257 ± 3.158	40.925 ± 4.148	0.012 *	0.233
CNFA (mm^2^/mm^2^)	0.0059 ± 0.0003	0.0057 ± 0.0003	0.607	0.078	0.0053 ± 0.0003	0.0057 ± 0.0003	0.004 *	0.123
CNFW (mm/mm^2^)	0.0213 ± 0.0002	0.0215 ± 0.0002	0.400	0.091	0.0222 ± 0.0003	0.0220 ± 0.0002	0.368	0.060
CNFrD	1.4638 ± 0.009	1.4684 ± 0.006	0.801	0.072	1.4331 ± 0.010	1.4500 ± 0.009	0.004 *	0.229
Dendritic cells (n/frame)	4.9519 ± 1.810	2.3541 ± 1.252	0.039 *	0.209	3.1908 ± 0.549	2.5806 ± 0.545	0.077	0.110
Neuroma (n/frame)	0.1984 ± 0.075	0.0519 ± 0.034	0.064	0.315	0.1465 ± 0.049	0.0692 ± 0.042	0.104	0.167
Axonal Beads (n/frame)	0.6694 ± 0.147	0.1669 ± 0.561	0.002 *	0.564	0.3890 ± 0.078	0.1278 ± 0.052	0.008 *	0.389

OSDI (Ocular Surface Disease Index); FBut (Fluorescein Break Up Time); SANDE (Symptom Assesment in Dry Eye); CNFD (Corneal Nerve Fiber Density); CNBD (Corneal Nerve Branch Density); CNFL (Corneal Nerve Fiber Length); CTBD (Corneal Total Branch Density); CNFA (Corneal Nerve Fiber Area); CNFW (Corneal Nerve Fiber Width); CNFrD (Corneal Nerve Fractal Dimension). Asterisks (*) in the table indicate statistical differences (*p* < 0.05).

**Table 3:** Effect size according to the subtype of DED.

	Evaporative Dry Eye		Aqueous Deficient Dry Eye	
	Standard Treatment	PRGF Treatment	Standard Treatment	PRGF Treatment
CNFD	0.366 ↓	0.288 ↑	0.263 ↑	0.413 ↑
CNBD	0.135 ↓	0.111 ↑	0.253 ↑	0.308 ↑
CNFL	0.402 ↓	0.162 ↑	0.243 ↑	0.322 ↑
Dendritic cells	0.130 ↓	0.432 ↓	0.258 ↓	0.054 ↓
Neuromas	0.141 ↓	0.117 ↑	0.282 ↓	0.321 ↓
Beadings	0.277 ↓	0.184 ↓	0.756 ↓	0.379 ↓
Break Up Time	0.096 ↑	0.052 ↓	0.274 ↓	0.464 ↑
Schirmer	0.521 ↓	0.261 ↓	0.272 ↑	0.379 ↑
OSDI Score	0.183 ↓	0.015 ↑	0.539 ↓	0.509 ↓
SANDE Frequency	0.661 ↓	1.398 ↓	0.663 ↓	0.874 ↓
SANDE Intensity	0.822 ↓	1.235 ↓	0.506 ↓	0.948 ↓

Arrows indicate the trend of the effect with respect to baseline values.

## Data Availability

All the obtained data used to support the findings of this study are available from the corresponding author upon reasonable request.
